# The right ventricle in congenital heart disease - Cardiac T1 mapping for measurements of diffuse myocardial fibrosis

**DOI:** 10.1186/1532-429X-18-S1-O25

**Published:** 2016-01-27

**Authors:** Nadya Al-Wakeel, Sarah Nordmeyer, Sevim Yilmaz, Sanaz Rastin, Frédéric H Münch, Felix Berger, Titus Kuehne, Daniel Messroghli

**Affiliations:** 1 grid.418209.60000000100000404Congenital Heart Disease / Pediatric Cardiology, Deutsches Herzzentrum Berlin, Berlin, Germany; 2 grid.418209.60000000100000404Deutsches Herzzentrum Berlin, Berlin, Germany

## Background

Measurements of myocardial extracellular volume (ECV) through cardiac magnetic resonance (CMR) based T1 mapping allow for non-invasive quantification of diffuse myocardial fibrosis. The evaluation of the right ventricle (RV), often mainly affected in congenital heart disease (CHD), is complicated by its typical morphologic characteristics - a thin myocardium and a distinct trabeculation. This study aims to evaluate ECV measurements of the RV in patients with CHD and to introduce a tool that simplifies RV ECV analysis.

## Methods

CHD patients (n = 47; mean age 27.9 ± 9.6 years) were prospectively enrolled and compared with 17 healthy volunteers (mean age 25.1 ± 2.7 years). T1 maps were generated with Modified Look-Locker Inversion recovery (MOLLI) T1 mapping in a single midventricular plane in short axis (SAX) and transverse orientation (TRANS) before and 15 minutes after bolus application of Gd-DOTA or Gd-DTPA. To measure ECV, T1 values of the anterior or inferior RV wall were used. Regions of interest (ROIs) were evaluated with regard to image quality (1: no artifacts in RV, good contrast between blood and myocardium; 2: minor artifacts, good/sufficient contrast; 3: major artifacts and/or insufficient contrast) and ROI thickness (1: > 2 pixels; 2: 1-2 pixels; 3: < 1 pixel). ECV from plane RV ROIs were compared with ECV obtained from a custom-made tool that derives the mean T1 values from a curved line manually drawn in the center of the myocardial wall ("centerline ECV").

## Results

RV ECV could be determined in 40 patients (32 ± 0.05) and 9 volunteers (0.28 ± 0.03) with a strong correlation of ECV values from SAX and TRANS (r = 0.91, and 0.95; p < 0.05, respectively). In both groups average image quality was rated with grade 1 and mean RV ROI thickness with grade 2. ECV could not be measured in cases of insufficient contrast between blood and myocardium or ROI thickness < 1 pixel. ECV from ROIs and corresponding centerline ECV correlated strongly in SAX and TRANS in CHD patients (r = 0.97, and 0.92; p < 0.05, respectively) and volunteers (r = 0.91; p < 0.05, respectively).

## Conclusions

1. RV ECV can be assessed with T1 mapping in SAX and TRANS provided that

a) image quality allows for sufficient distinction between blood and myocardium, and

b) RV wall thickness is > 1 pixel.

2. The application of a simple line centered in the RV myocardium instead of a plane ROI simplifies and accelerates measurements of RV ECV.

Under these conditions, measurements of RV ECV can be integrated into clinical CMR routine across a wide spectrum of CHD.

